# Patterns of health lifestyle behaviours: findings from a representative sample of Israel

**DOI:** 10.1186/s12889-022-14535-5

**Published:** 2022-11-17

**Authors:** Gabriel Nudelman, Sol Yakubovich

**Affiliations:** School of Behavioural Sciences, The Academic College of Tel Aviv-Yaffo, 6818211 Tel Aviv-Yaffo, Israel

**Keywords:** Health behaviours, Health lifestyle, Latent class analysis, Behavioural classes, Israel

## Abstract

**Background:**

Researchers are increasingly acknowledging the importance of understanding patterns of engagement in multiple, as opposed to isolated, health behaviours. Accordingly, several studies, targeting various geographical regions, have begun to identify and characterize unique classes of individuals in terms of their engagement in health behaviours, towards gaining insights that might guide tailored health interventions. Our study extends this stream of research to the Israeli context, as well as examines whether certain sociodemographic characteristics tend to be associated with affiliation to a specific group of individuals, i.e., class membership.

**Methods:**

Two large representative samples were obtained from the 2010 and 2017 National Social Surveys of the Israel Central Bureau of Statistics. Latent Class Analysis was used to identify different classes, based on levels of engagement in five health behaviours: fruit-and-vegetable consumption, physical activity, smoking avoidance, sleep, and influenza vaccination. Multinomial logistic regression was applied to test the associations between sociodemographic characteristics (age, gender, religion, education level) and class membership.

**Results:**

We identified three distinct classes (denoted “healthy”, “unhealthy”, and “mixed”). Overall, the “healthy” class was characterized by healthy levels of fruit-and-vegetable consumption and physical activity, a low rate of currently-smoking individuals, and a high proportion of influenza vaccination. The “mixed” class was characterized by healthy levels of fruit-and-vegetable consumption and sleep duration, moderate levels of physical exercise, a high proportion of currently light smokers, and a low rate of vaccination. The “unhealthy” class was marked by relatively low levels of engagement in health behaviours. Generally, older, Jewish, and more-educated individuals were more likely to belong to the “healthy” class, while young, non-Jewish, and less-educated individuals were more likely to belong to the “unhealthy” class. We further identified differences between the 2010 and 2017 samples (e.g., differences in men’s likelihood of being in the “unhealthy” class), suggesting that some behavioural patterns might have changed over time.

**Conclusion:**

This research identified three classes of engagement in health behaviours across two large and representative samples. Moreover, the classes were associated with unique sociodemographic characteristics. Consequently, our findings can highlight health-behavioural patterns relevant to different sub-populations that should be considered in public health interventions.

**Supplementary Information:**

The online version contains supplementary material available at 10.1186/s12889-022-14535-5.

## Background

Health behaviours, defined as activities for preventing and detecting diseases (i.e., voluntary screening of disease) or for enhancing health [1], are well known to have a critical influence on illness and life expectancy [2–4]. To achieve maximal health benefits, it is not enough to engage in a single health behaviour—it is necessary to engage in multiple such behaviours, which may potentially include synergetic effects [5]. Notably, however, most studies reporting health interventions have tended to target single behaviours (for a systematic review, see [6]), instead of attempting to simultaneously address multiple co-occurring behaviours.

In recent years, researchers and health practitioners have begun to depart from this tendency and to acknowledge the importance of documenting patterns of engagement in multiple (as opposed to single) health behaviours. Thus far, research in this vein, which has focused on people from the US, Germany, and Australia [7–9], has revealed that different populations demonstrate unique health behaviour patterns. These unique patterns are likely to be associated with distinct health outcomes, and to influence the effectiveness of various health promotion programs [9, 10].

The current study aims to contribute to this developing stream of literature by uncovering patterns of health behaviours in two representative samples of the Israeli adult population. To this end, we use a statistical methodology called Latent Class Analysis (LCA) to identify unique classes of individuals that display distinct patterns of engagement in five focal health behaviours: consumption of fruit and vegetables; physical activity, smoking avoidance, sleeping, and influenza vaccination. We further seek to understand whether specific sociodemographic characteristics (namely, age, gender, religion, and education) tend to be associated with membership in a particular class, as such an understanding can highlight sub-optimal patterns of engagement in health behaviours among sub-populations, and guide interventions that address these sub-populations.

### Health behaviours

The current study focuses on five key health behaviours that have been shown to have significant roles in improving health and decreasing morbidity and death. Moreover, these behaviours were measured in two large representative samples of the Israeli population, and have been found to contribute to classification of individuals into distinct classes characterised by different levels of engagement in health behaviours [8, 9]. The first is consumption of a healthy diet, and specifically, *sufficient consumption of fruits and vegetables*; this behaviour may prevent some types of cancer, such as colorectal, breast, lung and stomach cancer [11], as well as cardiovascular diseases, in addition to reducing blood cholesterol [12, 13]. The second is *physical activity*, which has been associated with positive effects on physical, mental and cognitive health [4, 14]. The third is *smoking avoidance*, which differs from the previous two behaviours in that its essence is avoidance of a behaviour that is known to have a negative impact on health. Cigarette smoking is related to coronary heart disease, high blood pressure, stroke and congestive heart failure [15]. The fourth behaviour is *adequate sleep*. Sleeping less than 7 or more than 8 h has been associated with poorer health and premature mortality, as compared with sleeping between 7 and 8 h [9], with excessively short (as opposed to excessively long) sleep duration being associated with more severe health consequences [16]. The fifth health behaviour is *influenza vaccination*. High rates of death and hospitalization due to influenza are reported annually worldwide [17–19]. In particular, influenza is responsible for approximately 801,200 reported infections, 4130 hospitalizations, and 1140 deaths annually in Israel [20]. Influenza vaccination reduces illness and mortality by an estimated 30-60%.

### Classifying individuals according to levels of engagement in multiple health behaviours

Recent findings suggest that some health behaviours are particularly likely to co-occur with one another [21, 22], and recent works have suggested conceptualizing health behaviours as a network, in which connections are determined on the basis of co-occurrence [23]. Co-occurrence of health behaviours can have health implications beyond the cumulative effects of engagement in individual health behaviours; for example, an individual’s likelihood of multimorbidity decreases as the number of health behaviours that he or she engages in increases [24]. Thus, researchers are increasingly seeking to understand patterns of engagement in multiple health behaviours, and to identify how these patterns differ across various sub-populations—towards developing tailored intervention programs that address these sub-populations on the basis of their needs.

Past studies have used LCA to classify individuals from various populations according to their levels of engagement in health behaviours [8, 9]. For example, a study conducted on a nationally representative sample of US adults revealed seven distinct classes of individuals, characterized by different patterns of health-promoting-, health-compromising- and discordant behaviours. The analysis in that study was based on information regarding smoking status, alcohol use, physical activity, physician visits, and flu vaccination [9]. Another study, conducted in Germany, identified three distinct classes: A healthy class and two heterogeneous classes [8]. A study conducted in Australia also identified three classes, labelled “moderate lifestyle”, “active poor sleepers”, and “poor lifestyle” [7].

To further identify behavioural patterns that can inform decision makers of suboptimal engagement in health behaviours among the population, as well as enable comparisons of these patterns between sub-groups and across countries, it is important to examine diverse populations of different cultures. Consequently, the first goal of the current study was to apply a similar analytic approach to a representative sample of the Israeli adult population, in order to uncover classes (unique patterns) based on different levels of engagement in the aforementioned five health behaviours: consumption of fruits and vegetables, physical activity, smoking avoidance, sleeping, and influenza vaccination. While an earlier study conducted in Israel has identified categories of health lifestyle behaviour, it entailed a non-representative sample of women aged 50 to 74, and used a different analytical technique [25].

### Sociodemographic characteristics associated with engagement levels in health behaviours

Since populations differ in their ability or motivation to adhere to health recommendations, the second goal of the current research, following classification of the sample, was to predict class membership according to sociodemographic variables, and specifically, gender, age, religion, and education level. Several studies have produced similar characterizations. For example, in a study based in Australia, individuals who were categorized as members of a class representing poor lifestyle behaviours were more likely than those in a relatively healthy class to be younger, to have lower socioeconomic status, and to have completed only primary/secondary education [7].

More generally, beyond the explicit focus on LCA-generated class membership, several studies have robustly linked individual sociodemographic characteristics with the likelihood of engagement in specific health behaviours. In studies focusing on *gender differences*, for example, women were more likely than men to report eating fruits, whereas men engaged more in physical activity [26, 27]. Gender differences have also been observed in sleeping patterns, with women having longer sleep latency compared with men [28]. *Age* has also been linked to health behaviours; for example, adherence to influenza vaccination increases with age [27, 29]. Among studies considering *religious affiliation*, Conservative Protestant women were shown to have higher fat intake compared with Catholic women [30, 31]. Finally, a higher *education level* has been associated with lower smoking rates among women [32], as well as with a higher likelihood of changing health behaviours (e.g., smoking cessation and initiation of physical activity) after being diagnosed with hypertension [33].

Of particular interest to the current study are previous analyses of the Israeli population that link sociodemographic variables to the health behaviours considered herein. For example, religion/ethnicity has been linked to physical activity: Arab individuals (85% Muslim [34]) were less likely to engage in physical activity compared with Jewish individuals [35]. Men and people with higher education levels also displayed higher levels of physical activity [36]. In addition, smoking prevalence was higher among men compared with women, and particularly among Arab men compared with Jewish men [36, 37]. Healthy levels of fruit- and vegetable consumption were higher among Arab individuals than among Jewish individuals [36]. Higher education levels were also associated with higher consumption of fruits and vegetables [36]. To our knowledge, no data have been produced regarding associations between sleep duration or influenza vaccination and sociodemographic variables in the Israeli population.

In sum, the two main goals of the present study were as follows: (1) to create classes of individuals according to their patterns of engagement in five health behaviours: consumption of fruits and vegetables; physical activity, smoking avoidance, sleeping, and influenza vaccination; and (2) to examine class membership associations with four sociodemographic variables: age, gender, religion, and education. To achieve our goals, we used two large datasets collected in different years, which also enabled us to examine whether changes in health behaviour classes occurred over time.

## Methods

### Participants and Procedure

This study analysed two representative samples that the Israel Central Bureau of Statistics (CBS) collected for its National Social Survey in 2010 (sample 1 [38]) and in 2017 (sample 2 [39]). The CBS’ National Social Survey is an annual survey that aims to assess the well-being and living conditions of the Israeli adult population. Inclusion criteria include residents who lived in Israel for at least six months and a minimum age of 20 years. Exclusion criteria include prisoners, residents in therapeutic institutions (such as nursing homes and hospitals for patients with chronic diseases), Israeli citizens that lived abroad for more than a year during the survey, immigrants and diplomats who lived in Israel for less than six months, and other residents that live outside recognized settlements [40]. The CBS operates in accordance with rules of ethics [41], ensuring both confidentiality of information received and availability of data and results to the public. The survey contains a core set of questions that remain constant over the years and that address various life domains such as education and religion. In addition, each survey contains a unique set of sections that the CBS uses to gather data regarding specific topics of interest, which change annually. The surveys at the focus of the current study were selected because they were devoted to health and lifestyle topics, and thus contained information regarding respondents’ health behaviours. Questionnaires were administered in participants’ homes in one of three languages: Hebrew, Arabic, or Russian [38].

Participants were sampled from the civil registration of the Israeli Ministry of Interior. In each survey, the final planned sample size was 7,500 respondents. The sampling method was stratified sampling using 86 sub-groups of the population based on combinations of sociodemographic variables (for further details regarding the sampling method, see [38]).

Sample 1 included a total of 7,524 participants, and sample 2 included a total of 7,230 participants. After exclusion of participants due to missing data, the LCA performed in the current research included 7,091 participants in sample 1 and 6,441 in sample 2. Sociodemographic variables for both samples are presented in Table 1. The samples were composed mostly of Jewish adults (20–44 years), who completed high school or achieved an academic degree. Estimated mean (± *SD*) of age was 44.9 (± 16.6) years for sample 1 (2010) and 45.9 (± 16.7) for sample 2 (2017), based on mid-category values as mean estimates for the age of each respected category (and lower boundary for the highest category). To conduct multinomial regression analysis (described further on), additional participants were excluded due to missing values in at least one sociodemographic variable, which resulted in a total of 6,980 individuals in sample 1 and 6,431 in sample 2.

**Table 1:** Sociodemographic Characteristics of Sample 1 and Sample 2 used in LCA

	Sample 1 (*N* = 7,091)	Sample 2 (*N* = 6,441)
Gender (men)	3444 (48.6%)	3172 (49.2%)
Age (in years)		
20–44	3768 (53.1%)	3295 (51.2%)
45–64	2216 (31.2%)	1959 (30.4%)
65+	1107 (15.6%)	1187 (18.4%)
Religion^a^		
Jewish	5682 (80.1%)	4936 (76.7%)
Muslim	891 (12.6%)	987 (15.3%)
Christian	232 (3.3%)	227 (3.5%)
Druze	135 (1.9%)	132 (2.1%)
Atheist or other religion	150 (2.1%)	155 (2.4%)
Education level^a^		
Lower than high school	1209 (17.3%)	1057 (16.4%)
High school	2481 (35.5%)	2237 (34.8%)
Non-academic secondary certification	1343 (19.2%)	1055 (16.4%)
Academic diploma	1948 (27.9%)	2086 (32.4%)

^a^Percentages were calculated after exclusion of participants that did not provide a response: Regarding religion, 1 and 4 participants were excluded in Sample 1 and Sample 2, respectively; regarding education, 110 and 6 participants were excluded in Sample 1 and Sample 2, respectively

### Measures

#### Fruit- and vegetable consumption

We assigned each participant a fruit-and-vegetable-consumption score with one of three values—healthy, unhealthy, or moderately healthy—on the basis of participants’ responses to questions about such consumption in their respective surveys. In sample 1, participants rated the extent to which they usually ate fruits and vegetables on a 4-point scale (1 – to a very great extent; 2 – to a great extent; 3 – to little extent; 4 – not at all); we categorized ratings of 1 and 4 as healthy and unhealthy eating levels, respectively, and ratings of 2 and 3 as moderately healthy eating. In sample 2, participants were asked to quantify the number of fruits and vegetables they usually consume per day, and were provided with examples of serving sizes (e.g., an apple, a cucumber). This measurement allowed for a more precise classification, as follows: healthy eating: 5 or more servings per day; moderately healthy eating: 2 or more but fewer than 5 servings per day; unhealthy eating: fewer than 2 servings per day. These thresholds are in accordance with previous studies [42].

#### Physical activity

In each sample, participants were asked various questions regarding the quantity and types of physical activity they engaged in weekly. After receiving relevant explanations, participants were asked about their engagement in vigorous-intensity exercise (activity that causes a lot of sweating and a large increase in heart rate, such as running) and in moderate-intensity exercise (activity that causes light sweating and a small increase in heart rate, such as walking). In line with previous studies [7], we translated these responses into a measurement of physical activity with one of three values: no physical activity (respondents who declared they did not exercise at all), insufficiently active (respondents who engaged weekly in 0 to 149 min of moderate-intensity activity or in 0 to 74 min of vigorous-intensity activity), or sufficiently active (respondents who engaged weekly in at least 150 min of moderate-intensity activity or 75 min of vigorous-intensity activity, respectively).

#### Cigarette smoking

Participants were asked whether they smoke nowadays, at least once a week (i.e., current smoker), and if not, whether they have smoked in the past or not. Consistent with previous studies [9], participants’ responses regarding their smoking habits were translated into a smoking score with five values: never smoked, former smoker, currently light smoker (up to 10 cigarettes a day), currently moderate smoker (between 11 and 20 cigarettes a day), or currently heavy smoker (21 or more cigarettes a day).

#### Sleep

Participants’ responses to questions regarding their sleeping habits (during the past month) were translated into a score with three values, based on research regarding healthy sleep duration [9]: shorter (than healthy) sleep duration (less than 7 h), healthy sleep duration (7–8 h), or longer sleep duration (more than 8 h).

#### Influenza vaccination

We assigned each participant a dichotomous vaccination score (i.e., yes or no), according to respondents’ answers regarding whether they had been vaccinated for influenza in the last 12 months [9, 43].

#### Sociodemographic variables

Gender was defined as male or female. Age was measured as an ordinal variable and divided into three categories: young adults (20–44 years), adults (45–64 years), and older adults (65 + years). Religion was categorized into three groups: Jewish (74.2%), Muslim (17.8%) and other (8%) [44]. Education was measured as the highest diploma received and coded into four levels: less than high school, high school (graduated with or without matriculation), post-secondary non-academic (such as diploma studies or certificate program), and academic (e.g., B.A., M.A., Ph.D.).

### Latent class analysis (LCA)

LCA is a procedure used to identify subgroups characterized by multiple dimensions. This procedure assumes that the population is composed of a (given) finite number of comprehensive and mutually exclusive groups of individuals, and that these groups are distinguished by unobserved, i.e., latent, variables [45, 46]. Since a latent class model uses categorical indicators, no assumptions regarding the distribution of the indicators are required [47]. In order to reveal these classes, and given a target number of classes, LCA uses the observed data (in our case, engagement levels in the focal health behaviours) to probabilistically create groups that maximize the homogeneity of the individuals within each class. In our context, this means that, within each class of individuals identified by LCA, levels of engagement in the different health behaviours should be similar [9].

For this analysis, we used the software PROC LCA 1.3.2 developed for SAS version 9.4 [48]. In our case, the target number of classes was unknown; accordingly, in line with previous studies [9], we estimated the optimal number of classes by comparing models with 1 to 10 classes according to the following fit statistic measurements [47]: Akaike Information Criterion (AIC), Bayesian Information Criterion (BIC), sample-adjusted BIC (SBIC), and Entropy. Lower values in AIC, BIC and SBIC indicate a better model fit. Higher Entropy values indicate higher certainty in the assignment of people to classes. Since our goal was to create distinguishable but meaningful classes, we also considered the interpretability of each model by examining the smallest probability of class membership and the class sample size [47].

### Multinomial logistic regression

To achieve the second goal of the study—prediction of class membership on the basis of sociodemographic variables (gender, age, religion and education level)—we conducted multinomial logistic regression, a generalization of logistic regression that includes more than two possible discrete outcomes. We selected this approach because, as elaborated in the Results section and in line with previous studies [9], our LCA procedure identified more than two classes of participants. IBM SPSS Statistics for Windows, version 23.0 (IBM Corp., Armonk, NY: USA), was used for this analysis.

## Results

Overall weighted proportions of the health behaviours, as well as of sociodemographic characteristics, are presented in the columns titled “Overall” in Table 2 (sample 1) and in Table 3 (sample 2). In general, most participants in both samples ate a moderate degree of fruits and vegetables, did not exercise, never smoked, and slept 7 to 8 h or less. Moreover, about a quarter of the participants reported getting the influenza vaccine in the previous 12 months.

**Table 2:** Proportions of Health Behaviors and Sociodemographic Variables by Class, 2010 Social Survey (*n* = 7,091; Sample 1)

		Overall	1- Healthy	2- Mixed	3- Unhealthy
Fruit- and Vegetable Consumption	Unhealthy	0.05	0	0	0.22
	Moderate	0.69	0.67	0.68	0.74
	Healthy	0.25	0.33	0.31	0.04
Physical Activity	None	0.50	0.35	0.41	0.88
	Insufficient	0.28	0.36	0.34	0.04
	Sufficient	0.22	0.28	0.25	0.07
Smoking	Currently light	0.12	0.03	0.23	0.12
	Currently moderate	0.11	0.01	0.09	0.29
	Currently heavy	0.03	0	0	0.13
	Past smoker	0.18	0.39	0	0.04
	Never	0.56	0.56	0.67	0.41
Sleeping	Shorter duration	0.51	0.66	0.13	0.73
	Healthy	0.45	0.31	0.85	0.19
	Longer duration	0.04	0.03	0.03	0.08
Influenza Vaccine	Yes^a^	0.22	0.45	0	0.11
**Sociodemographic variables**					
Gender	Men	0.49	0.51	0.41	0.55
Age	20–44	0.54	0.40	0.70	0.57
	45–64	0.31	0.35	0.25	0.33
	65 +	0.15	0.24	0.05	0.10
Religion	Jews	0.81	0.88	0.74	0.76
	Muslim	0.12	0.06	0.17	0.08
	Other	0.07	0.05	0.09	0.16
Education level	Lower than high school	0.17	0.16	0.13	0.25
	Secondary school completion	0.35	0.29	0.41	0.38
	Post-secondary non-academic	0.19	0.21	0.17	0.17
	Academic	0.28	0.33	0.28	0.18

^a^Have received influenza vaccination over the past 12 months

**Table 3:** Proportions of Health Behaviors and Sociodemographic Variables by Class, 2017 Social Survey (*n* = 6,441; Sample 2)

		Overall	1- Healthy	2- Mixed	3- Unhealthy
Fruit- and Vegetable Consumption	Unhealthy	0.14	0.03	0.19	0.24
	Moderate	0.49	0.42	0.29	0.73
	Healthy	0.37	0.55	0.52	0.03
Physical Activity	None	0.50	0.11	0.52	1
	Insufficient	0.31	0.55	0.30	0
	Sufficient	0.19	0.34	0.18	0
Smoking	Currently light	0.12	0	0.46	0.03
	Currently moderate	0.08	0	0.15	0.13
	Currently heavy	0.02	0	0.02	0.06
	Past smoker	0.18	0.3	0	0.16
	Never	0.6	0.7	0.38	0.62
Sleeping	Shorter duration	0.46	0.47	0.45	0.44
	Healthy	0.49	0.51	0.46	0.48
	Longer duration	0.06	0.02	0.08	0.08
Influenza Vaccine	Yes^a^	0.26	0.33	0.07	0.29
**Sociodemographic variables**					
Gender	Men	0.48	0.49	0.52	0.45
Age	20–44	0.53	0.47	0.65	0.51
	45–64	0.30	0.31	0.28	0.30
	65 +	0.17	0.21	0.07	0.19
Religion	Jews	0.78	0.87	0.75	0.68
	Muslim	0.15	0.07	0.16	0.24
	Other	0.07	0.06	0.09	0.08
Education level	Lower than high school	0.16	0.09	0.14	0.27
	Secondary school completion	0.36	0.32	0.44	0.35
	Post-secondary non-academic	0.16	0.16	0.17	0.15
	Academic	0.32	0.42	0.25	0.23

^a^Have received influenza vaccination over the past 12 months

### Identifying the number of classes for LCA: model goodness of fit

Fit statistics measurements for 10 models are summarized in Supplementary Tables S1 and S2 for samples 1 and 2, respectively. The lowest AIC values were achieved by the 5-class model in sample 1 and by the 8-class model in sample 2. However, in both samples, these models also had relatively low probability of membership (i.e., classes including very few people) and higher values of BIC and SBIC compared with other models. Moreover, the differences between some of the classes were minute. Therefore, these models were not considered optimal. Conversely, the BIC and SBIC values were lowest for the 3-class model in both samples, with reasonable representation of individuals in each class. Entropy, in our case, was not indicative of fit, as it displayed similar values for most models across samples. On the basis of these results, we selected the 3-class model for each sample, given its better overall fit indices, sufficient sample-size per class, and interpretable results.

### Characterization of the LCA classes according to health behaviour patterns

Proportions of degree of engagement in health behaviours by class are presented in Tables 2 and 3 for sample 1 and sample 2, respectively. In each sample, we labelled the three classes “healthy”, “mixed” and “unhealthy”, based on their corresponding patterns of engagement in health behaviours. Specifically, in each sample, the “healthy” class (as compared with the other classes) was characterized by the highest rate of fruit- and vegetable consumption, the highest proportion of sufficient physical activity (and the lowest proportion of no activity), a high proportion of people who were not currently smoking, and the highest proportion of vaccinated people. The healthy classes in the two samples differed in terms of their sleep duration patterns. In sample 2, sleep duration patterns in the healthy class were in line with the overall healthy tendencies observed for this class. Specifically, the majority of participants in the healthy class (51%) indicated healthy sleep duration, and this proportion was the highest proportion of individuals with healthy sleep duration across all classes. In sample 1, however, a high proportion of individuals in the healthy class (66%) indicated shorter-than-healthy sleep duration (less than 7 h). Moreover, only a third of the sample displayed healthy sleeping behaviour—a proportion substantially lower than that observed for the “mixed” class (85%).

On the other extreme, the “unhealthy” class (as compared with the other classes) displayed the highest proportions of individuals who indicated unhealthy fruit- and vegetable consumption, no physical activity, and currently-heavy smoking (in sample 1, the unhealthy class was also characterized by the highest proportion of currently-moderate smoking). Again, findings regarding sleep duration differed between samples. In sample 1, a large majority of individuals in the unhealthy class (73%) demonstrated shorter-than-healthy sleep duration, and this proportion exceeded that corresponding to any other class. In sample 2, though a large proportion of individuals indicated shorter-than-healthy sleep duration (44%), a larger proportion indicated healthy sleep duration (48%). In both samples, the proportion of vaccinated people was lower in the unhealthy class than in the healthy class, but it was higher than that in the “mixed” class.

The “mixed” class can best be characterized by mixed trends of engagement in health behaviours. In sample 1, most individuals in this class consumed fruits and vegetables to a moderate extent, whereas in sample 2, the majority of individuals in the “mixed” class displayed healthy eating (notably, this pattern was also observed in the “healthy” class). Regarding sleep, in sample 1, the vast majority of individuals in the “mixed” class (85%) demonstrated healthy sleep duration; this proportion was substantially higher than that corresponding to either of the other two classes. In sample 2, the proportions of participants demonstrating healthy and shorter-than-healthy sleep duration were roughly equal (46% and 45%, respectively) and did not differ starkly from those of the other classes. Regarding physical activity, in both samples, the predominant level of physical activity in the “mixed” class was not exercising at all. In addition, in both samples, the “mixed” class was characterized by the highest proportion of currently-light smokers, and in sample 2, this class was characterized by the highest proportion of currently-moderate smokers. Finally, in both samples, the “mixed” class displayed the lowest proportion of vaccinated individuals compared with the other classes.

### Class membership by sociodemographic variables

Proportions of sociodemographic variables by class are presented in Tables 2 and 3 for samples 1 and 2, respectively. Due to the large sample sizes, all the differences between the classes related to sociodemographic variables were statistically significant (*p* < .001) in both samples (see Supplementary Tables S3 and S4, for samples 1 and 2, respectively).

**Fig. 1:**
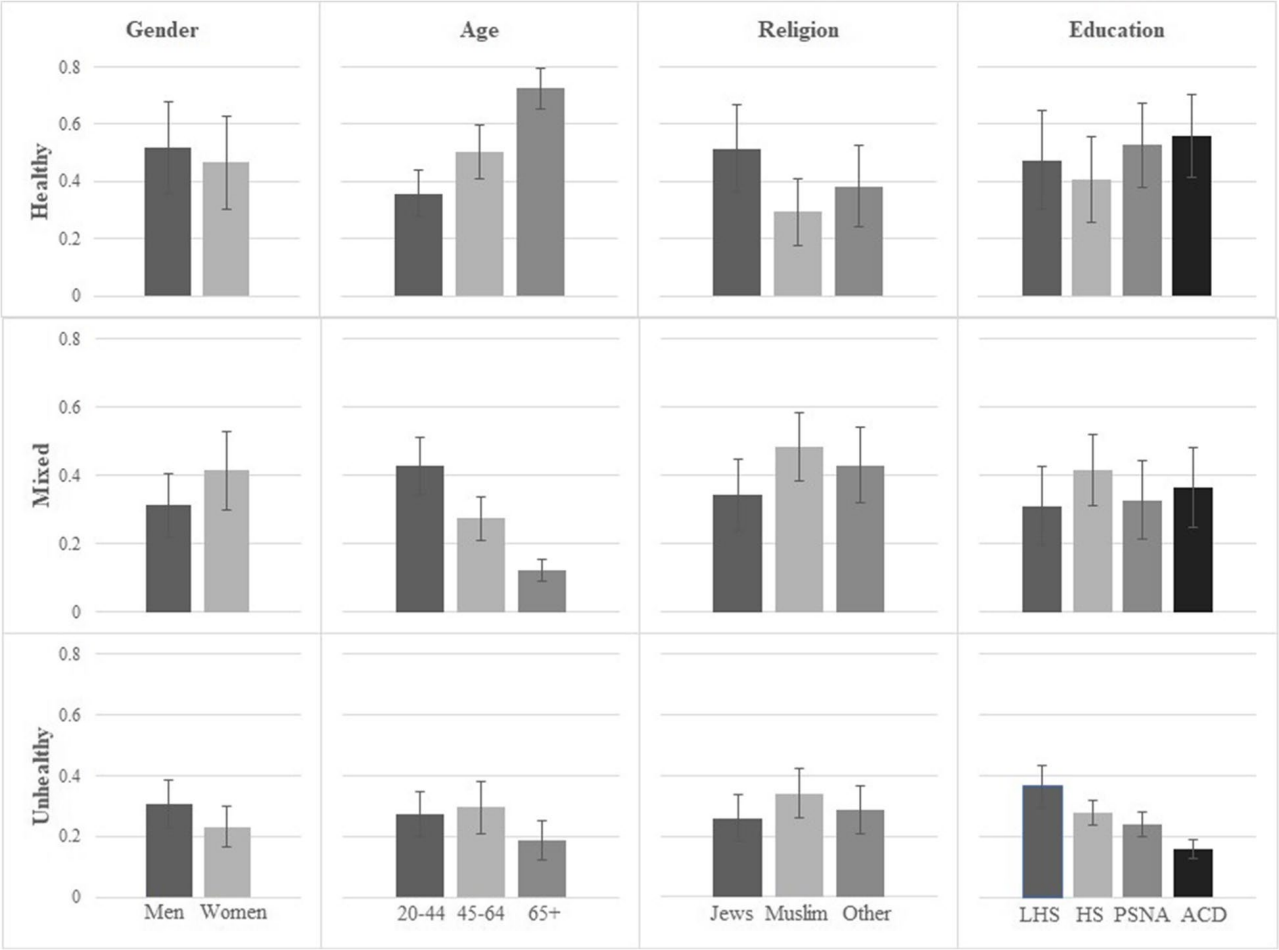
Probabilities and standard deviations of class membership by sociodemographic variables, 2010 survey Note. Each row represents a class and each column represents a sociodemographic variable. Abbreviations: LHS Lower than high school; HS High school; PSNA Post-secondary non-academic education; ACD Academic

**Fig. 2:**
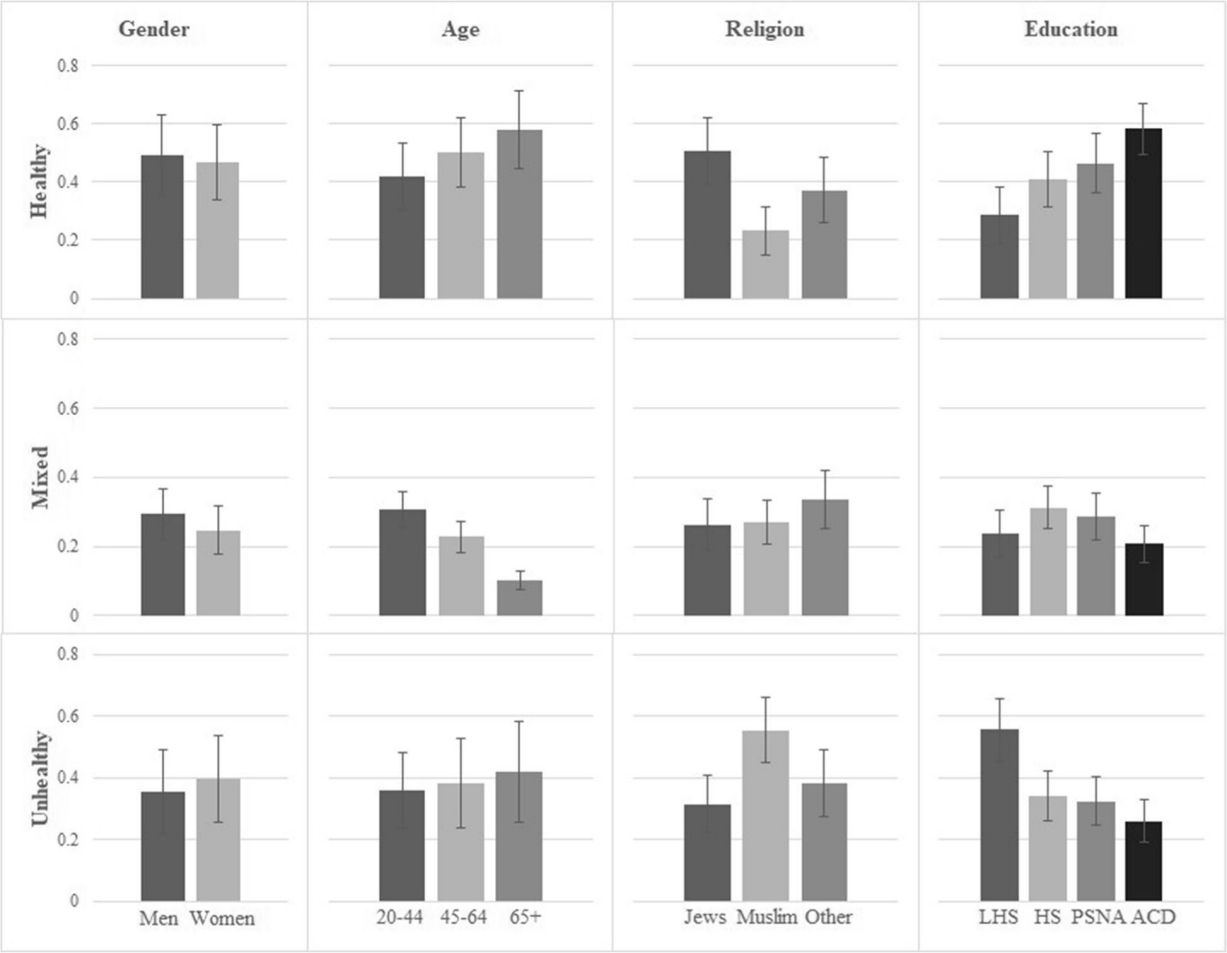
Probabilities and standard deviations of class membership by sociodemographic variables, 2017 survey Note. Each row represents a class and each column represents a sociodemographic variable. Abbreviations: LHS Lower than high school; HS High school; PSNA Post-secondary non-academic education; ACD Academic

Probabilities and standard deviations of class membership by sociodemographic variables are presented in Fig. 1 (for sample 1) and in Fig. 2 (for sample 2). In both samples, the probability of membership in the “healthy” class increased with age, and young adults had the highest probability of being members of the “mixed” class. With regard to religion, Jewish individuals had a higher likelihood to belong to the “healthy” class compared with members of other religions. Compared with the other religions, Muslims had the highest probability to be associated with the “mixed” class in sample 1, and the highest probability to be members of the “unhealthy” class in sample 2. Regarding gender, men had a higher probability of being members of the “unhealthy” class in sample 1, whereas women had a higher probability of being members of the “unhealthy” class in sample 2. Finally, the probability of being a member of the “unhealthy” class decreased as an individual’s education level increased, while the results concerning the “healthy” and “mixed” classes were inconsistent.

## Discussion

The analysis presented herein enabled us to uncover three distinct classes of individuals—denoted “healthy”, “mixed”, and “unhealthy”—that were characterized by different patterns of engagement in five health behaviours of interest (fruit- and vegetable consumption, physical activity, sleep duration, smoking avoidance, and influenza vaccination). The clear and interpretable classes are consistent with previous studies that found groups of individuals with healthy and unhealthy engagement levels in health behaviours [7, 9, 42, 49]. Moreover, this result is aligned with the findings of an earlier study conducted in Jerusalem that entailed a non-representative sample of women, which identified (using a different analytical technique) three categories of individuals, denoted “health promoting”, “inactive”, and “ambivalent” [25]. We further established connections between various sociodemographic characteristics (age, religion, gender, and education level) and an individual’s likelihood of belonging to a particular class. These findings, too, were aligned with those of a previous study of the Israeli population that found associations corresponding with our findings regarding the relationships between sociodemographic factors, such as gender and religion, and three health behaviours: fruit- and vegetable consumption, physical activity, and smoking [36]. We discuss our results in detail in what follows.

### Three classes of individuals with differing levels of engagement in health behaviours

In both samples we analysed, members of the “healthy” class tended to consume healthy quantities of fruits and vegetables; to engage in sufficient physical exercise; and to be non-smokers. Moreover, this class was characterized by the highest proportion of influenza-vaccinated individuals (as compared with the other two classes). The only difference between the healthy classes of sample 1 (from 2010) and of sample 2 (from 2017) was that only the latter tended to be characterized by healthy sleep duration, whereas the former tended to have shorter-than-healthy sleep durations. Class membership characteristics may contribute towards explaining this difference. Specifically, though the two samples were similar along many of the sociodemographic characteristics examined (e.g., in both samples the healthy class was associated with a relatively even proportion of males and females, Jewish religious affiliation, and high education levels, i.e., academic level), they differed somewhat in terms of age: Compared with the healthy class in sample 2, the healthy class in sample 1 was associated with lower proportions of young adults and higher proportions of older adults. Prior studies have suggested that healthy sleep durations may differ between age groups, with older individuals requiring less sleep (e.g., 6 h) [50]. Thus, for the generally older individuals in the “healthy” class of sample 1, the tendency for a shorter sleep duration may actually have represented “healthy” behaviour.

In both samples, the “mixed” class was characterized by a combination of different levels of engagement in health behaviours: moderate and healthy levels of fruit- and vegetable consumption and healthy sleep duration, but low engagement in physical activity, unhealthy levels of smoking, and low rates of influenza vaccination. However, the “mixed” class of sample 2 was characterized by higher proportions of light and moderate smoking compared with that of sample 1. Again, sociodemographic variables may contribute towards interpreting this difference: In general, in both samples, the “mixed” class was mainly associated with younger adults (20–44 years) and less educated individuals (mostly high school level). However, there were gender differences between the two samples: men were more likely to be associated with the mixed class in sample 2 compared with sample 1. Thus, our results may indicate that younger men are at increased risk of becoming smokers, a notion that follows previous findings linking gender with smoking [37].

Finally, in the two samples, the “unhealthy” class was mostly characterized by moderate consumption of fruits and vegetables, no physical exercise, smoking to a moderate or heavy extent, a substantial proportion of individuals with shorter-than-healthy sleep duration, and an intermediate likelihood of influenza vaccination (i.e., between the proportions of the “healthy” and “mixed” classes). In both samples, younger and less educated people were particularly likely to belong to the unhealthy class, but the two samples differed in terms of gender and religious affiliation. These differences are discussed in further detail in the following subsection.

### Behavioural patterns associated with sociodemographic sub-groups

Our findings regarding the relationships between sociodemographic variables and class membership can provide broader insights regarding the health risks faced by specific segments of the population, and they may also reveal ways in which these risks might have changed over time. In what follows we will provide two salient examples.

First, we found that the “unhealthy” class was characterized by a higher proportion of men in sample 1 (2010), but a higher proportion of women in sample 2 (2017). The high proportion of men in sample 1 may be explained by prior studies linking aspects of masculinity with engagement in unhealthy behaviours, such as smoking and saturated fat intake [51]. In turn, the decrease in this proportion seven years later may be attributable to a relatively recent trend for Israeli men—to a greater extent than women—to undergo medical examinations for early detection of malignant diseases, including faecal occult blood tests and colonoscopies [39]. Such examinations are likely to expose men to professional feedback regarding their health status, potentially increasing their motivation to behave in healthier ways and to modify risky behaviours.

Second, we observed a relationship between class membership and religious affiliation. Specifically, being Muslim predicted membership in the “unhealthy” class in sample 2 and membership in the “mixed” class in sample 1. These results are consistent with previous findings that Israeli Muslim individuals are at higher risk, compared with Israeli Jewish individuals, of engaging in unhealthy behaviours, such as smoking [37], not exercising [35], using less specialist care [52], and utilizing fewer early detection mammogram tests among women [53].

### Implications

This research examined health behaviour classes in the Israeli population by analysing two national databases, established at different time points. The rigorous data collection techniques used to create these samples suggest that our findings are likely to constitute a faithful representation of health behaviours in the Israeli population and in sub-groups within this population. As such, our findings can provide researchers, clinicians, and health decision makers with reliable insights regarding patterns of engagement in multiple health behaviours.

The clear and interpretable classes are consistent with previous studies that found groups of individuals with healthy and unhealthy engagement levels in health behaviours [7, 9, 42], and attest to the significance of LCA as an analytic technique for identifying meaningful groups related to health behaviours. Moreover, the identification of specific demographic characteristics related to each class supports the utility of such a classification, and highlights the need to consider individuals’ social context when developing and administering health-related evaluations and interventions.

Our results are compatible with a mechanism proposed to underlie global health inequalities [54]: the higher risk of disadvantaged populations of developing unhealthy lifestyle behaviours (e.g., Muslim individuals compared to Jewish individuals in our sample). The fact that an objective analysis approach revealed distinct classes of individuals engaged in “healthy” versus “unhealthy” patterns of multiple health behaviours, coupled with the fact that class membership could be predicted according to sociodemographic variables, suggests that disparate levels of engagement in health behaviours may explain health gaps across sociodemographic groups. These findings can inform decision makers in public health about the specific behavioural risks of different groups in the population, and enable them to adjust interventions to the particular needs of each sub-population.

Likewise, our findings may contribute to the development of health messages that target *multiple* health behaviours, and that are tailored according to the risk factors corresponding to specific sub-populations. Such tailoring can improve the cost-effectiveness of interventions. For example, since young adults are mainly associated with the “mixed” class, health interventions targeting this particular age group should focus more on increasing levels of exercise and vaccination adherence, while decreasing light smoking. Previous findings have already suggested that addressing multiple (as opposed to single) health behaviours in interventions may result in greater health benefits [55], and that tailored interventions are associated with greater improvement in health behaviour compared with non-tailored interventions [56, 57].

### Limitations

Several limitations warrant mentioning. First, the survey was based on self-report data, which may have resulted in socially desirable responding; that is, participants may have tended to present a favourable image of themselves [58]. Moreover, the measurement of fruit- and vegetable consumption was not identical across the two surveys, which might decrease the accuracy in the comparison of the two samples. However, our data were composed of large representative samples of the population, and were taken from surveys administered by an official governmental organization, which may have reduced the impact of such bias. Moreover, many of our findings were aligned with results from previous studies, lending further support to the validity of our results and conclusions.

Second, LCA requires categorical indicators to create the different classes [47]. It is important to note that different cut-off points that are used to transform metric variables into categorical variables (e.g., recoding exercise levels as sufficient, insufficient or not at all) may affect the latent classes. Nevertheless, these cut-off points have clinical and applied value, and they were based on previous research and national recommendations [9].

Third, since the number of real classes in the population is unknown, an estimation of the best model in terms of optimal balance between model fit and parsimony was required [45]. In both samples, we evaluated the models’ goodness of fit using various measures, but the results of these measures were not consistent across all the models. For example, while BIC and SBIC had lower values in the 3-class model, indicating its superiority, AIC displayed better values for models with more classes, although the differences between the values of the different models were small. Moreover, Entropy was not indicative of model fit in our samples, as it remained similar across models and had overall low values, which may imply that additional indicators are needed to improve the discrimination between classes [59].

Finally, additional sociodemographic variables might be associated with classes of individuals characterized by different levels of engagement in health behaviours. For example, future studies might consider focusing on indicators of socioeconomic status, such as education, income and occupation [60]. Specifically, education was included in the current investigation and proved to be an important variable for understanding group differences. In a study concerning smoking behaviours, income was not associated with health risks related to smoking, while the health risks of unemployed individuals—particularly managers and professionals—were higher compared to employed individuals [60]. Consequently, occupation may represent a promising candidate to better understand differences between classes.

## Conclusions

The current research is the first to examine patterns of multiple health behaviours among Israeli adults, based on two large samples representative of the general population. We found evidence of three distinct classes, with two of them (the mixed and unhealthy classes) characterized by some levels of unhealthy engagement in health behaviours. The findings provide evidence for the tendency of health behaviours to co-occur (with individuals tending to engage, for example, in generally healthy or unhealthy levels of various behaviours), although incongruous behavioural patterns were observed as well. Moreover, the cumulative findings of this research and past studies [7, 8, 25] might indicate a fundamental pattern wherein engagement in lifestyle behaviours can be adequately represented across countries and populations by three classes: (relatively) healthy, unhealthy, and inconsistent. Furthermore, each class displayed distinctive associations with sociodemographic variables, enabling us to reveal sub-populations that were most likely to engage in healthy versus sub-optimal behavioural patterns. In particular, membership in the “healthy” class was associated with older, Jewish individuals, and the probability of belonging to the “unhealthy” class decreased as education level increased. Although men displayed higher likelihood of belonging to the “unhealthy” class in sample 1, women displayed higher likelihood of belonging to the “unhealthy” class in sample 2. These findings constitute a first step towards understanding demographic and environmental determinants of lifestyle behaviours, which may lead to the development of tailored interventions for improving health outcomes that target a unique combination of multiple behaviours in specific populations.

## Supplementary Information

**Additional file 1.** **Supplementary Table S1. **Goodness of fits statistics for LCA models with 1 to 10 classes, 2010 survey.

**Additional file 2.** **Supplementary Table S2.** Goodness of fits statistics for LCA models with 1 to 10 classes, 2017 survey.

**Additional file 3. SupplementaryTable S3.** Multinomial Logistic Regression of 2010. Odd Ratio (95% Confidence Interval).

**Additional file 4. SupplementaryTable S4.** Multinomial Logistic Regression of 2017. Odd Ratio (95% Confidence Interval).

## Data Availability

Data and materials available through the Israel Central Bureau of Statistics: research@cbs.gov.il.

## Abbreviations

LCA: Latent Class Analysis
CBS: Israel Central Bureau of Statistics
