# Supplementary material for: Patterns of health lifestyle behaviours: findings from a representative sample of Israel
Source: BMC Public Health. 2022 Nov 17;22:2099. doi: 10.1186/s12889-022-14535-5 (PMC9670447; doi:10.1186/s12889-022-14535-5)
Supplement: Supplementary file 1 — Additional file 1. Supplementary Table S1. Goodness of fits statistics for LCA models with 1 to 10 classes, 2010 survey. [file 12889_2022_14535_MOESM1_ESM.docx]

**Supplementary Table S1**

Goodness of fits statistics for LCA models with 1 to 10 classes, 2010 survey.

| Number of classes: | 1 | 2 | 3 | 4 | 5 | 6 | 7 | 8 | 9 | 10 |
| --- | --- | --- | --- | --- | --- | --- | --- | --- | --- | --- |
| AIC | 837.74 | 477.21 | 380.81 | 348.35 | 342.89 | 341.95 | 348.23 | 355.21 | 370 | 388.09 |
| BIC | 913.27 | 635.14 | 621.14 | 671.08 | 748.02 | 829.47 | 918.16 | 1007.54 | 1104.76 | 1205.21 |
| SBIC | 878.31 | 562.00 | 509.92 | 521.73 | 560.53 | 603.85 | 654.4 | 705.65 | 764.74 | 827.06 |
| Entropy | 1.00 | 0.32 | 0.31 | 0.36 | 0.42 | 0.42 | 0.48 | 0.47 | 0.6 | 0.44 |
| Smallest membership  Probability | 1.00 | 0.36 | 0.26 | 0.18 | 0.01 | 0.01 | 0.01 | 0.01 | 0.01 | 0.01 |
