# Supplementary material for: Patterns of health lifestyle behaviours: findings from a representative sample of Israel
Source: BMC Public Health. 2022 Nov 17;22:2099. doi: 10.1186/s12889-022-14535-5 (PMC9670447; doi:10.1186/s12889-022-14535-5)
Supplement: Supplementary file 2 — Additional file 2. Supplementary Table S2. Goodness of fits statistics for LCA models with 1 to 10 classes, 2017 survey. [file 12889_2022_14535_MOESM2_ESM.docx]

**Supplementary Table S2**

Goodness of fits statistics for LCA models with 1 to 10 classes, 2017 survey.

| Number of classes: | 1 | 2 | 3 | 4 | 5 | 6 | 7 | 8 | 9 | 10 |
| --- | --- | --- | --- | --- | --- | --- | --- | --- | --- | --- |
| AIC | 923.42 | 498.24 | 428 | 392.98 | 369.76 | 351.76 | 348.21 | 347.42 | 356.22 | 361.64 |
| BIC | 997.89 | 653.96 | 664.96 | 711.19 | 769.22 | 832.46 | 910.15 | 990.61 | 1080.66 | 1167.32 |
| SBIC | 962.94 | 580.88 | 553.74 | 561.84 | 581.73 | 606.84 | 646.4 | 688.72 | 740.64 | 789.17 |
| Entropy | 1 | 0.3 | 0.37 | 0.39 | 0.37 | 0.37 | 0.38 | 0.43 | 0.42 | 0.47 |
| Smallest membership  Probability | 1 | 0.49 | 0.25 | 0.13 | 0.12 | 0.11 | 0.08 | 0.05 | 0.01 | 0.05 |
