# Supplementary material for: Patterns of health lifestyle behaviours: findings from a representative sample of Israel
Source: BMC Public Health. 2022 Nov 17;22:2099. doi: 10.1186/s12889-022-14535-5 (PMC9670447; doi:10.1186/s12889-022-14535-5)
Supplement: Supplementary file 3 — Additional file 3. SupplementaryTable S3. Multinomial Logistic Regression of 2010. Odd Ratio (95% Confidence Interval). [file 12889_2022_14535_MOESM3_ESM.docx]

**Supplementary Table S3**

Multinomial Logistic Regression of 2010. Odd Ratio (95% Confidence Interval).

| Referent: Unhealthy Class | Healthy | Mixed |
| --- | --- | --- |
| Gender (Referent: Women) |  |  |
| Men | 0.92 (0.92-0.93) *** | 0.57 (0.57-0.57) *** |
| Age (Referent: 45-64 years old) |  |  |
| Young adults (20-44 years old) | 0.75 (0.74-0.75) *** | 1.57 (1.57-1.58) *** |
| Old age (65+ years old) | 2.58 (2.56-2.6) *** | 0.8 (0.79-0.81) *** |
| Religion (Referent: Jewish) |  |  |
| Muslim | 0.6 (0.6-0.61) *** | 1.31 (1.3-1.32) *** |
| Other | 0.7 (0.69-0.71) *** | 1.2 (1.19-1.21) *** |
| Education (Referent: Academic) |  |  |
| Other | 0.32 (0.31-0.32) *** | 0.35 (0.34-0.35) *** |
| Secondary education | 0.41 (0.41-0.42) *** | 0.63 (0.63-0.64) *** |
| Post-secondary education | 0.63 (0.62-0.63) *** | 0.6 (0.59-0.6) *** |

*** *p* < .001.
