# Supplementary material for: Patterns of health lifestyle behaviours: findings from a representative sample of Israel
Source: BMC Public Health. 2022 Nov 17;22:2099. doi: 10.1186/s12889-022-14535-5 (PMC9670447; doi:10.1186/s12889-022-14535-5)
Supplement: Supplementary file 4 — Additional file 4. SupplementaryTable S4. Multinomial Logistic Regression of 2017. Odd Ratio (95% Confidence Interval). [file 12889_2022_14535_MOESM4_ESM.docx]

**Supplementary Table S4**

Multinomial Logistic Regression of 2017. Odd Ratio (95% Confidence Interval).

| Referent: Unhealthy Class | Healthy | Mixed |
| --- | --- | --- |
| Gender (Referent: Women) |  |  |
| Men | 1.26 (1.25-1.26) *** | 1.32 (1.32-1.33) *** |
| Age (Referent: 45-64 years old) |  |  |
| Young adults (20-44 years old) | 0.86 (0.85-0.86) *** | 1.31 (1.3-1.32) *** |
| Old age (65+ years old) | 1.18 (1.18-1.19) *** | 0.41 (0.4-0.41) *** |
| Religion (Referent: Jewish) |  |  |
| Muslim | 0.33 (0.33-0.34) *** | 0.6 (0.6-0.61) *** |
| Other | 0.58 (0.58-0.59) *** | 1.08 (1.08-1.09) *** |
| Education (Referent: Academic) |  |  |
| Other | 0.22 (0.22-0.23) *** | 0.65 (0.65-0.66) *** |
| Secondary education | 0.54 (0.54-0.55) *** | 1.15 (1.15-1.16) *** |
| Post-secondary education | 0.62 (0.62-0.63) *** | 1.18 (1.17-1.19) *** |

*** *p* < .001
